# Mumps and Parotitis

**Published:** 1915-05-22

**Authors:** 


### MUMPS AND PAROTITIS.

The terms "mumps" and "parotitis" are sometimes used as though they were synonymous. This, however, is a mistake. Mumps is a generalised specific febrile disease which has swelling of the parotid glands as its most frequent and most prominent local symptom. But other organs may be involved in the inflammatory process, and sometimes they are involved prior to the parotid glands. Further, mumps has a very conspicuous epidemic character, and the parotid swelling rarely, if ever, suppurates. As opposed to all this a parotitis which tends to suppurate, and which is non-epidemic, occurs in various circumstances, and is no more true mumps than an inflamed tonsil is scarlet fever. There are, therefore, good reasons for keeping the two names separate and apart, seeing that they represent two quite distinct conditions. Even the term "epidemic parotitis" as applied to mumps cannot be defended, as it narrows unjustly the general character of the disease. Still, it is sometimes adopted, while the localised inflammations of the gland above referred to are grouped under the term "secondary parotitis." The fact is that the term "mumps" has come down to us from the good old days when our predecessors possessed the fortunate faculty of inventing names free from the contamination of pathological doctrine. Nowadays, forsooth, our names must needs display a pseudo-scientific quality, with, not infrequently, subsequent confusion. What term better than "mumps" could save us from the risks which attach themselves to a nomenclature based on a haughty and over-confident pathological speculation?

The general differences between mumps and a non-specific or secondary parotitis have been indicated above. In practice there need rarely be any diagnostic difficulty if one only remembers the circumstances in which the latter condition is apt to occur. Some years ago Mr. Stephen Paget collected records of a number of cases of parotitis in which the swelling of the parotid gland was associated with various abdominal and pelvic disturbances, and this particularly in patients who had been subjected to surgical operations. Further observations confirmed these records, but showed that parotitis was by no means restricted to operation cases, nor, for the matter of that, to abdominal lesions. Odd cases have been reported in such different conditions as suppressed menstruation, pernicious anæmia, fæcal tumour, and the use of a pessary. Putting these eccentricities on one side, it may perhaps be said that the three medical conditions in which parotitis is most likely to be met with are gastric ulcer or at least hæmatemesis, enteric fever, and pneumonia. An alternative is here put to gastric ulcer because in many of the cases there has been a sudden and free hæmorrhage, with few other gastric symptoms, and the patient has made a very rapid recovery. Hence the doubt may arise whether there really has been ulceration, or whether, on the other hand, the bleeding is to be explained in some other fashion. One writer describes parotitis in pneumonia as a most serious complication, but this is not in harmony with general experience. Whether complicating gastric ulcer or enteric fever or pneumonia, parotitis is certainly an annoying feature, and it may suppurate and lead to a good deal of local trouble, especially if it is bi-lateral; but to describe it as necessarily alarming is an over-statement of the position. Concerning all instances of the so-called secondary parotitis there exists a sharp division of opinion as to the mode of causation. One teaching is that the irritant reaches the gland through the blood, and that the inflammation is a local manifestation of a general blood infection. The opposing view is that the irritant proceeds from a septic buccal cavity and along Stenson's duct, the invasion being favoured by a suppressed or scanty flow of the parotid secretion, the result of febrile or other influences. Some few years ago, on one and the same evening, two different contributors to the Medico-Chirurgical Society read papers on the nature of "secondary parotitis," and they expressed with equal confidence diametrically opposing conclusions. Whatever may be thought of the dispute as a matter of doctrine, the local infection view seems to have had the chief influence on practice; arising from it, at least in part, is the inclination to pay a more considerable attention to the state of the mouth both after operations and in febrile illnesses.

In a recent study of mumps Dr. Anthony Feiling* draws attention to a feature of the disease which is not generally known. This is a slight increase in the leucocytes of the blood with a lymphocytosis which is both relative and absolute, the lymphocytosis being present on the first day of the disease and persisting for at least fourteen days. Such a blood picture may help to distinguish a case of true mumps from other swellings of the parotid or sub-maxillary gland. Further, it is of value in distinguishing orchitis as a complication of mumps from the same condition consequential on gonorrhœa, as in the latter the blood film shows a decided increase of the polymorphonuclear cells. The principal application of this test in practice would be in those rare cases of mumps in which the orchitis precedes the parotid swelling.

Another feature of Dr. Feiling's paper is the emphasis he places on the serious character of the occasional complications of mumps, as orchitis, pancreatitis, nephritis, and such conditions of the nervous system as meningitis, various paralyses, optic neuritis, and so on. In an article in The Hospital of April 24 allusion was made to the value of lumbar puncture in the diagnosis of cerebro-spinal meningitis. Much the same may be said in reference to mumps, in which a lymphocytosis of the cerebro-spinal fluid may be present even though there are no clinical signs of meningitis. It is not unimportant to recall that a disease which in the vast majority of instances pursues an uneventful course has, all the same, possibilities of mischief.

* *Quarterly Journal of Medicine*, April, 1915.

